# *Ammopiptanthus mongolicus* stress-responsive *NAC* gene enhances the tolerance of transgenic *Arabidopsis thaliana* to drought and cold stresses

**DOI:** 10.1590/1678-4685-GMB-2018-0101

**Published:** 2019-11-14

**Authors:** Xinyue Pang, Min Xue, Meiyan Ren, Dina Nan, Yaqi Wu, Huiqin Guo

**Affiliations:** 1 College of Life Sciences, Inner Mongolia Agricultural University, Hohhot, China.; 2 College of Medical Technology and Engineering, Henan University of Science and Technology, Luoyang, China.; 3 State Key Laboratory of Cotton Biology, Anyang, China.; 4 Key Laboratory of Desert and Desertification, Chinese Academy of Sciences, Lanzhou, Gansu, China.

**Keywords:** Ammopiptanthus mongolicus, expression, NAC, tolerance, transgene

## Abstract

Drought and cold are the primary factors limiting plant growth worldwide. The *Ammopiptanthus mongolicus NAC11* (*AmNAC11*) gene encodes a stress-responsive transcription factor. Expression of the *AmNAC11* gene was induced by drought, cold and high salinity. The AmNAC11 protein was localized in the nucleus and plays an important role in tolerance to drought, cold and salt stresses. We also found that differential expression of *AmNAC11* was induced in the early stages of seed germination and was related to root growth. When the *AmNAC11* gene was introduced into *Arabidopsis thaliana* by an *Agrobacterium*-mediated method, the transgenic lines expressing *AmNAC11* displayed significantly enhanced tolerance to drought and freezing stresses compared to wild-type *Arabidopsis thaliana* plants. These results indicated that over-expression of the *AmNAC11* gene in *Arabidopsis* could significantly enhance its tolerance to drought and freezing stresses. Our study provides a promising approach to improve the tolerance of crop cultivars to abiotic stresses through genetic engineering.

## Introduction

Abiotic stresses, such as drought and low temperatures, are the main stress factors affecting the growth and development of plants. Plants have evolved complex stress-tolerance mechanisms, including the perception of stress signals, the transduction of transmembrane signals, and the generation and transmission of endogenous signaling molecules, which leads to changes in the related genes, metabolic pathways, and even cellular structures, thereby protecting plant cells against damage by stresses (Anderson *et al.*, 1994; Fowler *et al.*, 1996; Chaves *et al.*, 2003). Over the past decades, thousands of genes and dozens of metabolic and signaling pathways have been identified in response to drought and/or cold environments (Flower and Thomashow, 2002; Xiong *et al.*, 2002; Rabbani *et al.*, 2003; Shinozaki *et al.*, 2003; Zhou *et al.*, 2007; Gimeno *et al.*, 2009; Hadiarto and Tran*,* 2011; Stolf-Moreira *et al.*, 2011).

Numerous studies have revealed that transcription factors (TFs) play important roles in the regulation of stress-related genes. Generally, TFs are molecules located the downstream of signal transduction pathways or at the nodes of different stress signaling pathways that confer stress tolerance to plants by regulating downstream gene expression (Hussain *et al.*, 2011; Huang *et al.*, 2012). Several TF families have been demonstrated to be crucial in plant stress tolerance, among which the plant-specific NAC family [(NAM (no apical meristem), ATAF (Arabidopsis transcription activation facto), CUC (cup-shaped cotyledon)] has been the focus of studies in recent years due to its significant roles in the responses and adaptation of plants to adverse environments, particularly drought, salt, cold and heat stresses (Kim *et al.*, 2007; Yoo *et al.*, 2007; Zheng *et al.*, 2009; Tran *et al.*, 2010; Hao *et al.*, 2011; Skirycz *et al.*, 2011; Pei *et al.*, 2013; Wang *et al.*, 2013; Mao *et al.*, 2014; Qin *et al.*, 2014).

The NAC proteins from different plant species typically possess a highly conserved NAC domain at the N-termini and a highly variable transcriptional activation region at the C-termini. The NAC domain contains approximately 150-160 amino acid residues, including at least five conserved regions (A-E) related to nuclear localization and interactions with target DNA elements in the promoter regions of their downstream genes (Ooka *et al.*, 2003). A recent study showed that NAC TFs function by forming homo- or heterodimers, and the interaction regions are primarily located in the NAC domain in the N-termini of these proteins, although some interaction regions have been located in the C-termini of a few NAC proteins (Jensen *et al.*, 2010). The diversified structures not only provide the basis for the extensive physiological functions of the NAC family but also demonstrate the complexity of these proteins with respect to their regulatory mechanisms.

An increasing number of studies have recently shown that some *NAC* genes have potential applications in crop stress-resistance modification by genetic engineering. The molecular regulatory mechanism by which *NAC* TFs mediate plant responses and resistance to abiotic stresses has been revealed to some extent. In the rose (*Rosa rugosa*), *RhNAC2* and *RhNAC3* could confer petal resistance to dehydration by regulating the expression of genes related to cell wall and osmotic processes, respectively (Jiang *et al.*, 2014). Banana (*Musa nanaLour.*) *MaNAC1* might be involved in the formation of cold tolerance in the banana fruit via interactions with the ICE-CBF (inducer of CBF expression - C-repeat binding factor) signal pathway (Shan *et al.*, 2014). Rice (*Oryza sativa*) *SNAC1*/*OsNAC9* and *OsNAC10* are probably involved in stress resistance, including roles in regulating stress responses, preventing cells from dehydration, detoxification, protecting proteins and other macromolecules, oxidation-reduction, and ion balance, thereby enhancing rice resistance to drought, high salinity, low temperature, etc. (Jeong *et al.*, 2010; Redillas *et al.*, 2012). *Arabidopsis ANAC019/NAC019, ANAC055,* and *ANAC072/RD26* are the first reported *NAC* genes involved in abiotic stress responses and are induced by drought, salt stress, and ABA (abscisic acid). Hence, these genes could improve drought resistance in transgenic overexpression lines (Tran *et al.*, 2004; Jensen *et al.*, 2010; Hickman *et al.*, 2013). Several other *NAC* genes in *Arabidopsis*, including *AtNAC2, LOV1 (light, oxygen, voltage1), ANAC096, JUB1 (jungbrunnen 1)* and *SHYG (speedy hyponastic growth),* also played important roles in the formation of stress resistance, such as low temperature, dehydration, salt, osmotic and oxidative stresses, heat, and flooding (He *et al.*, 2005; Mao *et al.*, 2007; Wu *et al.*, 2009; Jensen *et al.*, 2013; Saad *et al.*, 2013; Xu *et al.*, 2013). However, few studies have cloned these genes from strongly resistant plants. Most previous studies on *NAC* genes have been limited to gene cloning and expression analyses, accordingly, to examine the regulatory mechanism of *NAC* gene expression.

*Ammopiptanthus mongolicus* (Leguminosae) has strong stress resistance to cold, drought, salt, and alkali conditions, and this plant maintains leaves under harsh conditions, including cold winters and hot summers (-30 °C to 50 °C), annual precipitation of less than 200 mm, annual evaporation of greater than 3000 mm, gravelly or sandy soil, and salty and alkali soil. *A. mongolicus* is the only broad-leaved evergreen plant occurring in western Inner Mongolia and Ningxia, as well as part of the desert areas in Gansu. This plant provides excellent materials for the study of plant resistance mechanisms and the data mining of stress resistance genes. In recent years, a large number of genes related to stress resistance have been obtained from this plant by cDNA library construction, transcriptome sequencing, and expression profile analyses (Zhou *et al.*, 2012; Pang *et al.*, 2013; Liu *et al.*, 2013). In our previous work, two cold- and drought-induced *NAC* sequences, namely, *AmNAC4* and *AmNAC11*, were identified in the *A. mongolicus* transcriptome by using RNA-seq (Wu *et al.*, 2014).

In this study, the expression profiles/patterns of *AmNAC11* in response to various abiotic stresses and in different *A. mongolicus* plant organs were analyzed using semi-quantitative RT-PCR. The coding region of the *AmNAC11* gene was cloned and functional analyses were conducted in both transgenic *Arabidopsis* protoplasts and plants. This research not only provides important knowledge related to the expression regulation and resistance of *AmNAC11* and its mechanism of action, but also provides new insights and a basis for analyzing the molecular mechanisms of stress resistance to drought and cold in *A. mongolicus*. These results may provide genetic resources for the development of resistant crops via genetic engineering.

## Materials and Methods

### Plant materials and abiotic stress experiments

*A. mongolicus* seeds collected from Hohhot, Inner Mongolia, China were sterilized and soaked in water at 25 °C for 3-4 days and then cultured at 25 °C under a 16-h light/8-h dark cycle, according to Wu *et al.* (2014). One-and-a-half-month-old *A. mongolicus* plants were treated as follows: (1) for drought stress, the plants were subjected to natural drought at 25 °C (cultured at 25 °C under a 16-h light/8-h dark cycle without watering); (2) for cold stress, the plants were maintained at 4 °C in a low temperature-programmable incubator under dim light; (3) for salinity stress, the plants were dipped in 250 mM NaCl and maintained at 25 °C with a 16-h light/8-h dark cycle; (4) for heat stress, the plants were maintained in an incubator at 42 °C. At different time points (0, 2, 6, 12, 24 and 48 h), stressed *A. mongolicus* tissues were immediately frozen in liquid nitrogen. Three independent biological replicates were performed.

### Transformation of *Arabidopsis* and transgenic plant materials

The pMD19-T-*AmNAC11* plasmid was constructed by amplifying the entire coding region of *AmNAC11* by PCR with upstream *Xba*I and downstream *Sma*I linker primers and cloned into the *Xba*I/*Sma*I site of the binary vector pCAMBIA 3300. *Arabidopsis* plants were transfected with *Agrobacterium tumefaciens* strain GV3101 by vacuum infiltration (Bechtold *et al.*, 1993).

*Arabidopsis* seeds were vernalized at 4 °C for 3-4 days, and then cultured at 22 °C under a 16-h light/8-h dark cycle. 2-4weeks old *Arabidopsis* plants were subjected to the following treatments: (1) for drought stress, the plants were subjected to natural drought for 7 d; (2) for cold stress, plants were maintained at 4 °C in a low temperature-programmable incubator under dim light and maintained at -8 °C for 8 h under dim light. All vernalized seeds were cultured in 1/2 MS medium and subjected to the same treatments; stressed *Arabidopsis* tissues were immediately frozen in liquid nitrogen.

### DNA and RNA extraction

Genomic DNA was extracted from young leaves of *A. mongolicus* following the protocol in Sambrook and Russell (2001). Total RNA was extracted from the leaves, stems, roots, pods and flowers of *A. mongolicus* using TRIzol reagent. Purified RNA was treated with RNase-free DNase I (Takara, Dalian, China) prior to precipitation.

### Expression analysis

Approximately 1.5 μg of total RNA was reverse transcribed into cDNA using M-MLV reverse transcriptase (TaKaRa). The cDNA was amplified by PCR using the following primers: NAC11F: AATGCCACTCCCAATCTC AACAG; NAC11R: CCTTCAGTCTCGTGCTACCGTG. To standardize the results, the relative abundance of *Amactin* was also determined and used as the internal standard. PCR for expression analysis was performed with the following cycling profile: 94 °C for 3 min; 35 cycles at 94 °C for 30 s, 61 °C for 30 s, and 72 °C for 45 s; and a final extension for 10 min at 72 °C. Aliquots of the PCR reactions were loaded onto agarose gels and, after electrophoresis, stained with ethidium bromide.

### Gene cloning and protein analysis

Full-length cDNA was obtained by a 3’Rapid Amplification of cDNA Ends (3’RACE) protocol using the mRNA extracted from *A. mongolicus* as template (TaKaRa, Dalian, China). PCR for the cloning of *AmNAC11* was performed with the following cycling profile: 94 °C for 3 min; 30 cycles at 94 °C for 45 s, 63 °C for 45 s, and 72 °C for 1 min; and a final extension for 10 min at 72 °C. The deduced protein sequences were aligned using DNAMAN. A phylogenetic tree was constructed by MEGA5 using the Neighbor-Joining (NJ) method, followed by a bootstrap analysis of 1000 replications.

### Subcellular localization

The subcellular localization of the AmNAC11 protein was examined by adding the green fluorescent protein (GFP) to the end of the AmNAC11 protein via cloning to create a fusion protein. The entire coding region of the target gene was amplified by PCR and inserted into the *Xba*I and *Sma*I sites of the vector pBI 221. The recombinant plasmid, PBI221-*AmNAC11*-*GFP*, was introduced into *E. coli* DH5α. *Arabidopsis* transformation and selection was performed according to Du *et al.* (2011). The root tip cells of transformed *Arabidopsis* were observed using a laser confocal scanning microscope (Ti-U, Nikon, Japan).

### Expression analysis of AmNAC11-induced genes

Nine-day-old *Arabidopsis* seedlings grown on agar medium were transferred to agar medium with or without stress, and the expression levels of the genes were measured by semi-RT-PCR. The stress-inducible genes due to *AmNAC11* overexpression were compared using *RAB18*, *RD29A*, *RD29B*, *COR47*, *COR15A*, *COR15B*, *HSF* and *P5Sc* genes tested in drought stress, and *KIN*, *RD29A*, *COR47*, *COR15A*, *COR15B*, *HSF* and *P5Sc* genes tested in cold stress. The actin gene of *A. mongolicus* (*Amactin*) was used as a reference gene.

### Statistical analysis

All experimental data are reported as the average and standard deviation (SD) of three replicates, and statistical tests were conducted with SPSS v12.0 (IBM Corporation, New York, USA). Values are denoted as significant (*p* < 0.05) or highly significant (*p* < 0.01).

## Results

### Responses of *AmNAC11* to multiple abiotic stresses

**Figure 1:**
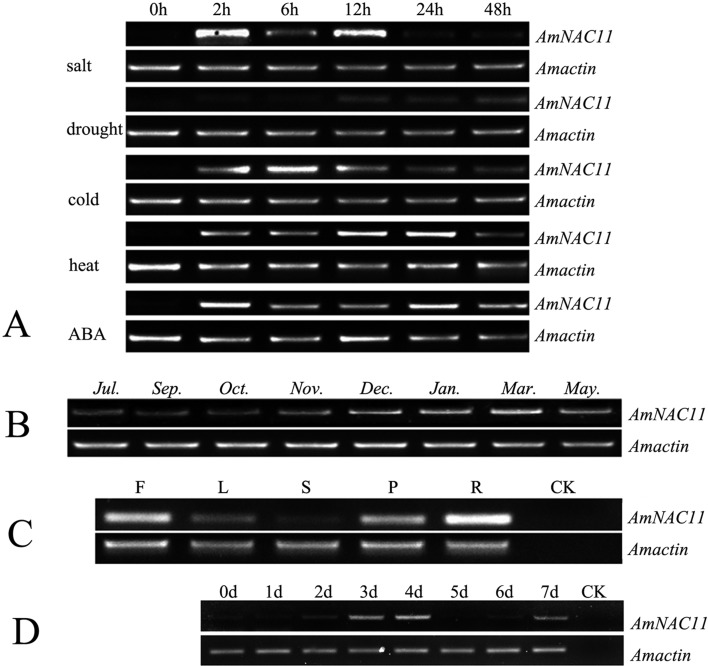
Variation in *AmNAC* gene expression. (A) *AmNAC11* gene expression in *Ammopiptanthus mongolicus* among indoor treatments; (B) *AmNAC11* gene expression in *Ammopiptanthus mongolicus* during field sampling; (C) *AmNAC11* gene expression in various organs. F: Flowers; L: Leaves; S: Stems; P: Pods; R: Roots; (D) *AmNAC11* gene expression in *Ammopiptanthus mongolicus* within 7 days of seed germination. (C and D) CK: the primers (as shown) were employed by PCR using double-steamed water to replace the cDNA as a blank control.

The effects of various abiotic stresses on *AmNAC11* expression were examined. For the freezing treatment, the expression of *AmNAC11* increased significantly after 2-6 h and then decreased gradually, but it was still higher than that of the untreated group after 48 h. For the drought treatment, a slight increase in expression was also observed in the middle stage (12 h), and the expression levels reached a maximum at 48 h. For the heat treatment, the expression of *AmNAC11* was consistently relatively higher than that of the 0 h control, especially at the early stage (Figure 1A).

The expression of *AmNAC11* was analyzed in *A. mongolicus* leaves at several key time points under natural conditions. The results showed that the expression of *AmNAC11* was significantly up-regulated at low temperatures (November to the next March) and was also expressed under drought stress (July) (Figure 1B), based on the meteorological data of Huhhot in 2014 (Table 1). These results indicated that *AmNAC11* responded to low temperature and drought stresses.

**Table 1:** Climatic index of Huhhot city of Inner Mongolia of China in 2014.

	Jan.	Feb.	Mar.	Apr.	May.	Jun.	Jul.	Aug.	Sep.	Oct.	Nov.	Dec.
Temperature (°C)	-10.9	-6	2.3	8	18	20.6	21.5	20.6	15.1	7.8	-1.5	-8.1
Relative humidity (%)	59	40	35	33	32	51	69	67	59	50	52	51
Precipitation (mm)	5.2	3.8	3.6	1.9	7.8	96	192.6	144	97.3	9	7.2	7
Sunshine duration (h)	174.2	202.4	253.8	257.1	265.7	209.9	230.9	224.7	215.3	238	171.3	186.5

### Expression of *AmNAC11* in various organs and at the seed germination stage

The leaves, stems, roots, flowers, and pods of *A. mongolicus* were collected from mature plants grown in the field. Based on a comparison of *AmNAC11* expression between tissue types for plants collected in the field in May (Normal growing condition, Figure 1B, C), *AmNAC11* expression was substantially higher in the roots than in other organs during this period. The plants were not affected by low temperature, drought, or other environmental stresses during sampling, suggesting that *AmNAC11* likely played an important role in plant growth, especially in root growth, and might exhibit some resistance to permeability-related stresses, such as drought and salt.

An increase in *AmNAC11* expression was observed within 7 days of seed germination in *A. mongolicus*, and the expression levels on days 1, 3, and 4 were higher than those in the control. Thus, *AmNAC11* might be involved in root growth, but not cotyledon development, during germination (Figure 1D).

### Characterization and protein prediction of the *AmNAC11* gene

**Figure 2:**
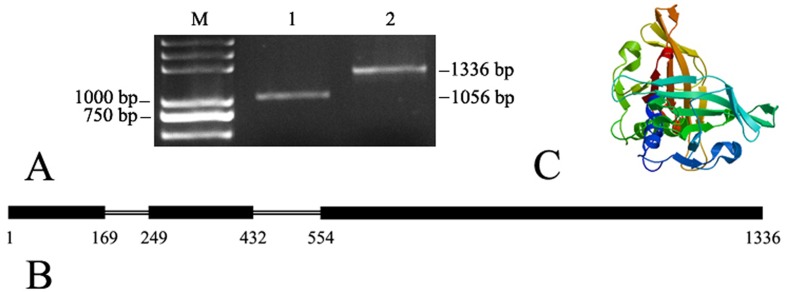
Structural analysis of the *AmNAC11* gene. (A) Electrophoretogram of *AmNAC11* (1. *AmNAC11* cDNA full-length 1056 bp; 2. *AmNAC11* genome DNA full-length 1336 bp); (B) structural diagram of *AmNAC11* genomic DNA; (C) predicted tertiary structure of the *AmNAC11* protein (Arnold *et al.*, 2006; Benkert *et al.*, 2011; Biasini *et al.*, 2014).

Using cDNA and genomic DNA of *A. mongolicus* as templates, amplified products were obtained with specific primers for full-length *AmNAC11* (Figure 2A). *AmNAC11* genomic DNA contained 3 exons and 2 introns (Figure 2B) and encoded a protein of 292 aa with a predicted isoelectric point of 6.54. The main secondary structure of the protein includes α-helix, β-sheet, β-turn, and random coil. The AmNAC11 secondary structure predicted using SOPMA (https://npsa-prabi.ibcp.fr/) (Geourjon and Deleage, 1995) (not shown) indicated that the proportion of random coils in the protein, which are involved in linking the other secondary structure elements, was high (53.77%). In addition, the main secondary structure elements were α-helices (28.42%) and β-sheets (13.01%). The sub-domains A and E mainly contained β-pleated sheets and α-helix structures, the sub-domains B and C primarily comprised β-pleated sheets, while sub-domain D contained α-helices, β-pleated sheets, and β-turns. The result of homologous modeling using SWISS-MODEL (Figure 2C) showed a few helical elements surrounding a twisted β-pleated sheet structure.

**Figure 3:**
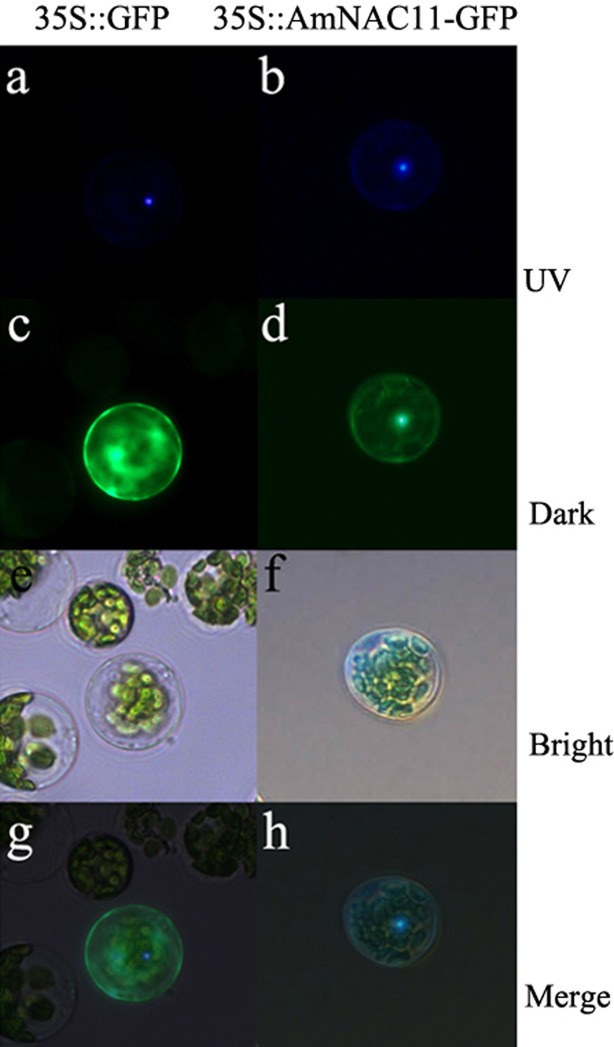
Subcellular localization of AmNAC11 in transgenic *Arabidopsis* protoplast cells. Cells were bombarded with constructs carrying GFP or AmNAC11-GFP. GFP and AmNAC11-GFP fusion proteins were transiently expressed under the control of the cauliflower mosaic virus 35S promoter in protoplast cells and observed with a laser scanning confocal microscope. Images are dark field (c, d), bright field (e, f), and combined (g, h), and UV field for DAPI nuclear stain (a, b).

Based on the subcellular localization analysis, in UV vision (Figure 3A, B), DAPI staining was used to show the location of the nucleus. The empty vector PBI221-*GFP* had no obvious localization in protoplast cells of *Arabidopsis thaliana* with green fluorescence in the nucleus, cytoplasm, and cell membrane (Figure 3C). After transformation of the recombinant plasmid PBI221-*AmNAC11*-*GFP* in protoplast cells of *A. thaliana*, green fluorescence was detected in the nucleus (Figure 3D). In bright field imaging (Figure 3E, F), the cells exhibited good growth conditions. The merged image (Figure 3G, H) confirmed that the AmNAC11 protein had a nuclear localization signal (NLS).

**Figure 4:**
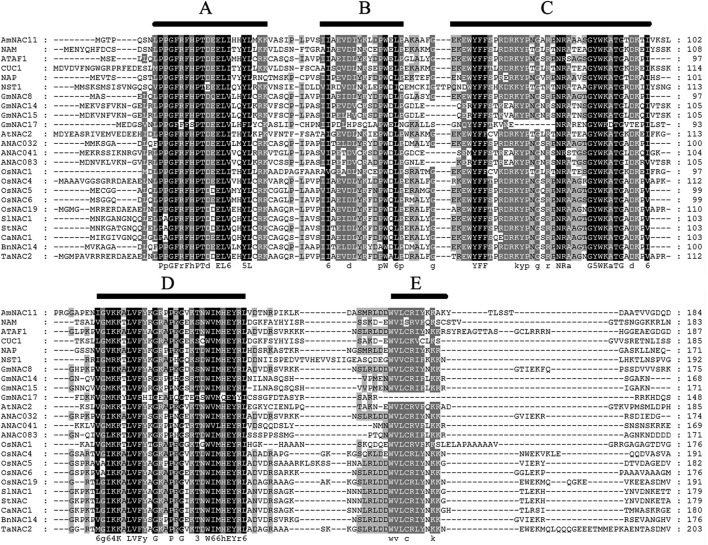
Alignment of AmNAC11 and NAC protein sequences of other species. ATAF1 (At1g01720), ANAC032 (NP177869), ANAC041 (NP001118435), ANAC083 (NP196822), AtNAC2 (At5g39610), CUC1 (AB049069), NAP (At1g69490), NST1 (At2g46770), OsNAC1 (AB028180), OsNAC11 (AB028183), OsNAC5 (AB028184), OsNAC6 (AB028185), OsNAC19 (AY596808), NAM (X92205), BnNAC14 (AY245886), GmNAC8 (EU661911), GmNAC14/GmNAC016 (EU661914), GmNAC15 (ACD39373), GmNAC17 (EU661917), TaNAC2 (AY625683), and CaNAC1 (AY714222).

An alignment generated using the online Clustal W2 tool (Figure 4) showed that the amino acid residues of the AmNAC11 protein at the N-terminus were highly conserved. Its structural domain comprised approximately 150 amino acid residues with high conservation, which could be further divided into 5 sub-domains, A, B, C, D, and E. The five sub-domains constituted the NAC structural domain, exhibiting typical structural characteristics of NAC transcription factors. The amino acids at the C-terminus were highly diverse, but a few relatively well-conserved amino acids, including proline (P), serine (S), and glutamate (E), were still detected in this region. Phosphorylation has a great influence on protein function, and the phosphorylation of protein kinase C plays an important role in metabolism, gene expression, cell differentiation, and proliferation. The phosphorylation of the AmNAC11 protein occurs mainly on serine (S) and threonine (T) according to an online NetPhos tool analysis (http://www.cbs.dtu.dk/services/NetPhos/).

### *AmNAC11* transgenic plants showed increased abiotic stress resistance

To explore the function of AmNAC11 in planta, we developed transgenic *Arabidopsis* constitutively expressing *AmNAC11* genes under the control of the 35S promoter. Semi-quantitative RT-PCR was used to detect the transcripts of *AmNAC11* in the homozygous overexpression plants. Four representative homozygote lines (AmNAC11-1, AmNAC11-2, AmNAC11-3 and AmNAC11-4) with high expression levels were confirmed (Figure S1). Two of them (AmNAC11-1 and AmNAC11-2) were used in the following experiments. No notable morphological differences were observed between the wild-type and transgenic plants throughout their life cycle.

The wild-type and transgenic plants were exposed to different abiotic stresses, including low temperature, drought, and salt, to determine whether *AmNAC11* is involved in plant defense against abiotic stress. Phenotypic differences among treatments and the defensive response function of this gene were examined.

### Drought stress

**Figure 5:**
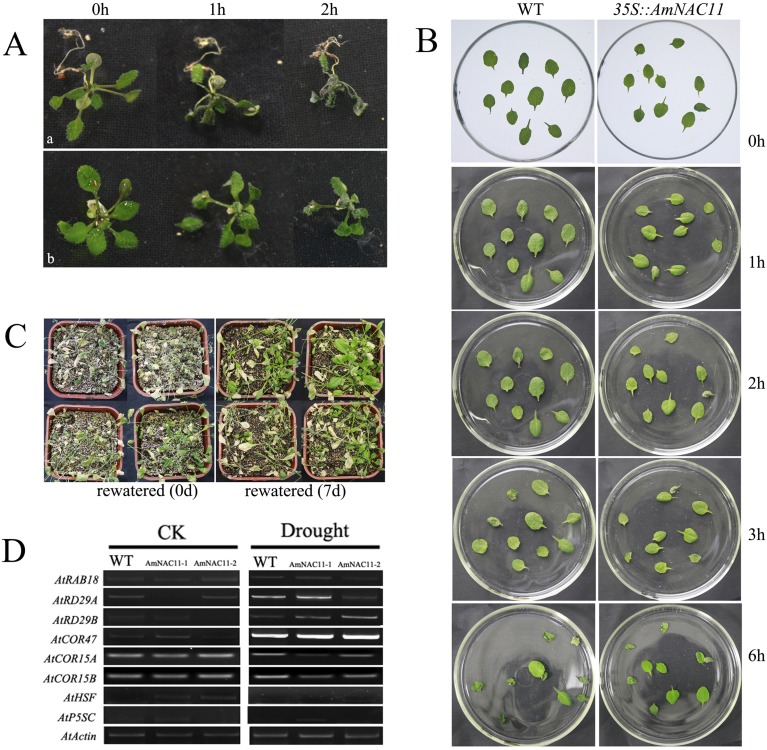
Phenotypes of *AmNAC11* transgenic plants with increased resistance to drought stress. Drought stress tolerance analyses of *AmNAC11* transgenic *Arabidopsis* plants. (A) Above-ground plant part drought tolerance analysis of *AmNAC11* transgenic *Arabidopsis* plants. Drought stress: 2-week-old transgenic and wild-type *Arabidopsis* were randomly harvested at 25 °C; (B) leaf drought tolerance analysis of *AmNAC11* transgenic *Arabidopsis* plants. Drought stress: 3-week-old wild-type and transgenic plant leaves were treated without watering for 1, 2, 3 and 6 h. The growth status of treated leaves is shown (a, the wild-type plants; b, the transgenic plants); (C) drought tolerance analysis of *35S::AmNAC11* transgenic *Arabidopsis* plants. (D) The drought-related gene expression in AmNAC11 transgenic *Arabidopsis* plants, CK, all the plants cultured at 25 °C under 16-h light/8-h dark cycle without drought stress treatment.

To examine resistance to drought stress, wild-type and transgenic plants were cultured under similar growth conditions. Within 1-3 hours, the leaves of transgenic and wild-type plants showed significantly different degrees of wilting after natural drying at room temperature for 8 h (Figure 5A). In addition, the above-ground plant parts obtained from transgenic and wild-type *Arabidopsis* with similar growth at 2 weeks of age were randomly harvested and placed in empty Petri dishes under the same conditions, and the extent of wilting was observed. The wilting degree of *A. mongolicus AmNAC11* transgenic plant leaves was significantly less than that of wild-type plants in *vivo* (Figure 5C and Figure S2A). The result shows that the weight loss rate of line AmNAC11-1 is 52.24A 2.33% and that of line AmNAC11-2 is 50.46w 1.28%, otherwise the rate of WT is 58.74c 2.89%, in the first 3 h of treatment, suggesting that AmNAC11 increases the water retention capacity of leaves.

In *vitro* (Figure 5B and Figure S2B, C), the plants cultured for 4 weeks under normal conditions were stopped watering for drought treatment, the results showed that the leaves of wild-type plants showed obvious wilting and drying phenotypes after about 15 days of water deprivation, however, only some transgenic plants showed similar symptoms, and most of the leaves of transgenic lines remained green and alive. The survival rate was 3.7 ± 0.9% of wild-type plants after resuming watering 5 days, while the survival rates of transgenic lines AmNAC 11-1 and AmNAC11-2 were as high as 53.2 ± 4.1% and 61.4 ± 5.8%, respectively.

Since AmNAC11 is a transcription factor, the drought-inducible gene (*RAB18*, *RD29A*, and *RD29B*) expression in transgenic plants was much higher than that in wild-type *Arabidopsis* without drought treatment, and the expression of *COR47 COR15A*, *COR15B*, *HSF* and *P5Sc* genes was the same. After 8 h of drought treatment, the expression of *RD29B* in transgenic lines was higher than that in wild-type *Arabidopsis*. The expression of *RD29A*, *COR15A* and *COR15B* in the transgenic lines was slightly lower than that in wild-type plants. And the expression of the other genes was no significant difference between transgenic and wild-type *Arabidopsis* (Figure 5D).

### Low-temperature stress

**Figure 6:**
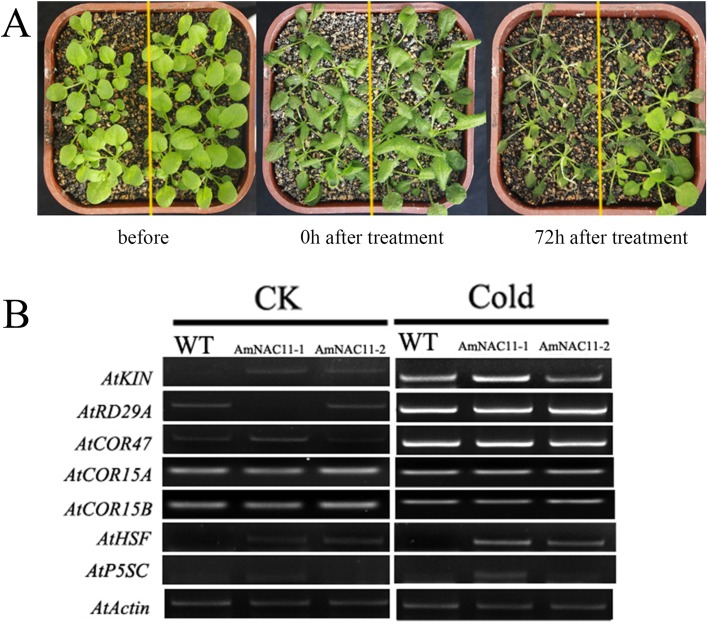
Phenotypes of *AmNAC11* transgenic plants with increased resistance to freezing stress. (A) Cold stress tolerance analyses of *AmNAC11* transgenic *Arabidopsis* plants. (B) The low temperature-related gene expression in AmNAC11 transgenic *Arabidopsis* plants. CK, all the plants cultured at 25 °C under 16-h light/8-h dark cycle without low-temperature stress treatment.

Because the *AmNAC11* gene showed high expression under low-temperature stress (Figure 1A), we compared wild-type and transgenic plants under low-temperature stress. The growth status of wild-type and transgenic plants cultured for 4 weeks was similar prior to the freezing treatment. After treatment at -8 °C for 8 h, the two plant types showed different degrees of leaf wilting. After 10 days of recovery, the wild-type plants almost died; except for damage and wilting on individual leaves, most of the transgenic seedlings had normal appearances and quickly recovered normal growth (Figure 6A and Figure S3). The survival rate of wild type was only 2.72 ± 0.33%, while those of transgenic lines AmNAC11-1 and AmNAC11-2 were 49.741.58% and 57.38 ± 3.26% respectively, and the plant heights were 4.62 ± 0.43 cm and 4.78 ± 0.32 cm respectively, which are significantly higher than those of wild type (0.69 ± 0.47 cm) (Figure S4). These results showed that *AmNAC11* could significantly increase the freezing resistance of transgenic *Arabidopsis*.

In the low-temperature-inducible gene expression analysis, in the CK groups, the gene expression of *KIN*, *HSF* and *P5Sc* in transgenic plants was much higher than that in wild-type *Arabidopsis*, the *RD29A* gene expression in transgenic plants was lower in AmNAC11-1 and AmNAC11-2 than in wild-type *Arabidopsis*, and the gene expression of *COR47* was up-regulated in transgenic plants compared to that in wild-type plants. Strikingly, the expression of all of the genes was up-regulated after cold-treatment for 8 h in wild-type and transgenic *Arabidopsis* plants except *KIN*-*HSF* and *P5CS* (Figure 6B).

## Discussion

Hao *et al.* (2010) found a transcriptional repression domain consisting of 35 amino acids in the D sub-domain of the DNA binding domain in NAC in soybeans. This repression domain was named NARD (NAC Repression Domain). Since then, NARD-like sequences, containing 17 residues (G**K*LVFY*G**P*G*K**W*MHEYRL) with 12 conserved amino acids (GKLVFYPWMHER), have also been found in other NAC proteins. The results of the amino acid sequence analysis in this study showed that the AmNAC11 transcription factor contained similar sub-domain D sequences, i.e., GVKKALVFYKGRPPKGVKTNWIMHEYRL. Moreover, the sequences LVFY and MHEYRL were highly conserved (Figure 4). Therefore, we inferred that sub-domain D of the abiotic stress-related NAC transcription factor also contained the transcriptional repression domain.

Hao *et al.* (2010) proposed that NAC contained both NARD and activation domains, and the tolerance ability of plants under abiotic stresses depended on the relative strengths of NARD and the activation domain. Putative nuclear localization sequences have been detected in the C and D sub-domains of many NAC domains. Tran *et al.* (2004) found that the RD26 contained a nuclear localization signal, and the NAC domain was essential for the entrance of RD26 into the nucleus. A GFP-RD26 fusion protein was localized in the nucleus, and RD26 lacking the NAC domain was localized in both the cytoplasm and nucleus. Lu *et al.* (2007) found that ATAF1 is located in the nucleus and the nuclear localization sequence was localized in the sub-domain D.

In this study, the AmNAC11 transcription factor was localized to the nucleus (Figure 3D), and the sequence GVKKALVFYKGRPPKGVKTNWIMHEYRL in its sub-domain D (Figure 4) might play a particularly important role in nuclear entry and subsequent functions.

The NAC transcription factors are not only a relatively large protein family in plants but also a specific transcription factor family in these organisms. In our laboratory, we constructed a full-length cDNA library for *A. mongolicus* in the previous works and obtained the NAC transcription factor family through plasmid sequencing and Blastn alignment. Some *AmNAC* transcription factor genes involved in stress resistance were screened using a digital gene expression analysis. The present results showed that high expression of *AmNAC11* was induced by both drought and low-temperature stresses and was also induced by salt and heat to some extent. These results indicated that *AmNAC11* might induce broad-spectrum resistance to multi-abiotic stress.

The effect of drought stress on the growth of young plant cells is manifested in the growth of the root system. In this study, transgenic *Arabidopsis* overexpressing the *AmNAC11* gene could resist drought stress at the beginning of germination, indicating that *AmNAC11* may be involved in the response to drought stress at the germination stage by promoting plant root growth. This conclusion was consistent with the higher expression of *AmNAC11* in *A. mongolicus* roots.

Freeze-sensitive plants typically do not absorb melting water back into the protoplast as temperatures increase, resulting in dehydration of the protoplasm and dried tissues. For freezing injuries, cell membrane damage affects the lipids on the membrane and destroys protein structure. *AmNAC11* transgenic *Arabidopsis* not only had better freezing resistance compared with that of wild-type plants but also exhibited less leaf wilting in response to the freezing injury. This result indicated that in the freeze dehydration process, transgenic plants could maintain cell integrity. This phenomenon further indicated that the AmNAC11 protein might promote the expression of membrane skeleton-related proteins.

The results of this study proved that *AmNAC11* could respond to drought and low-temperature stress and effectively improve the resistance of transgenic plants under these stresses. Currently, more and more publications reported that NAC TFs interact with other proteins, such as calcium-dependent protein kinases, WRKY, MYB, and ATDOF5.8, to participate in the plant stress response (He *et al.*, 2015; Zeng *et al.*, 2015; Shan *et al.*, 2016), but the *NAC* gene expression regulatory mechanism is poorly understood. Its regulatory mechanism and signaling pathway will be the focus of future research. The present results not only revealed the expression regulation and stress resistance function of *AmNAC11* and its potential regulatory mechanism, but also provided further insight into the molecular mechanism of resistance to drought and cold stresses in *A. mongolicus* and other plants.

## Supplementary material

The following online material is available for this article

Figure S1 The expression of *AmNAC11* in the transgenic plants.

Figure S2 The morphological analyses of wild type or transgenic plant leaves.

Figure S3 Tolerance analyses of *AmNAC11* transgenic *Arabidopsis* plants ten days after cold stress.

Figure S4 Evaluation of cold resistance of *AmNAC11* transgenic *Arabidopsis* lines.

*Associate Editor: Adriana S. Hemerly*
