# Supplementary material for: Ammopiptanthus mongolicus stress-responsive NAC gene enhances the tolerance of transgenic Arabidopsis thaliana to drought and cold stresses
Source: Genet Mol Biol. 2019 Nov 14;42(3):624–34. doi: 10.1590/1678-4685-GMB-2018-0101 (PMC6905445; doi:10.1590/1678-4685-GMB-2018-0101)
Supplement: Supplementary file 2 [file 1415-4757-GMB-42-3-2018-0101-suppl2.pdf]

Supplementary Material to "*Ammopiptanthus mongolicus* stress-responsive *NAC* gene enhances the tolerance of transgenic *Arabidopsis thaliana* to drought and cold stresses"

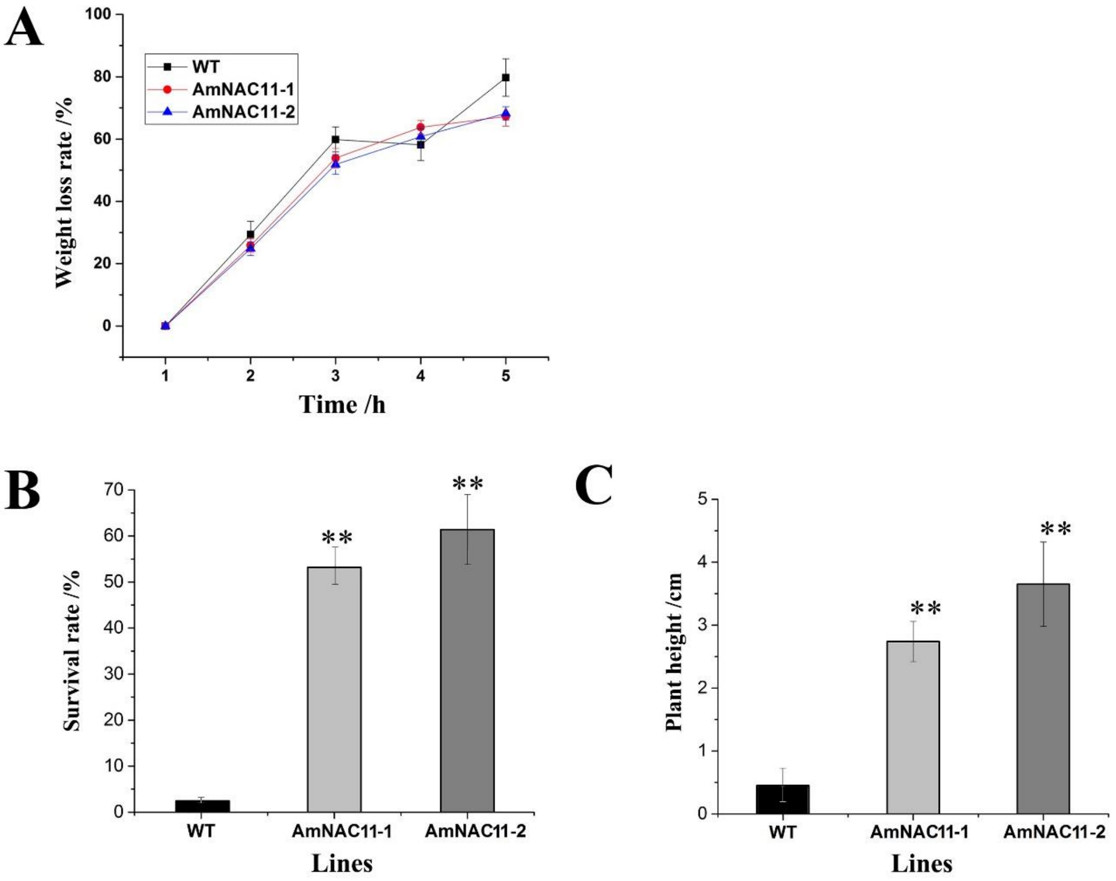

Figure S2 – The morphological analyses of wild type or transgenic plant leaves. (A) Water loss rates of the detached leaves; (B) survival rates of the wild type and transgenic lines on the 5th day of rewatering (** p<0.05); C, Plant heights of the wild type and transgenic lines on the 5th day of rewatering (** p<0.05).
