# Supplementary material for: Ammopiptanthus mongolicus stress-responsive NAC gene enhances the tolerance of transgenic Arabidopsis thaliana to drought and cold stresses
Source: Genet Mol Biol. 2019 Nov 14;42(3):624–34. doi: 10.1590/1678-4685-GMB-2018-0101 (PMC6905445; doi:10.1590/1678-4685-GMB-2018-0101)
Supplement: Supplementary file 3 [file 1415-4757-GMB-42-3-2018-0101-suppl3.pdf]

Genetics and Molecular Biology, 42, 3 (2019)

Supplementary Material to "*Ammopiptanthus mongolicus* stress-responsive *NAC* gene enhances the tolerance of transgenic *Arabidopsis thaliana* to drought and cold stresses"

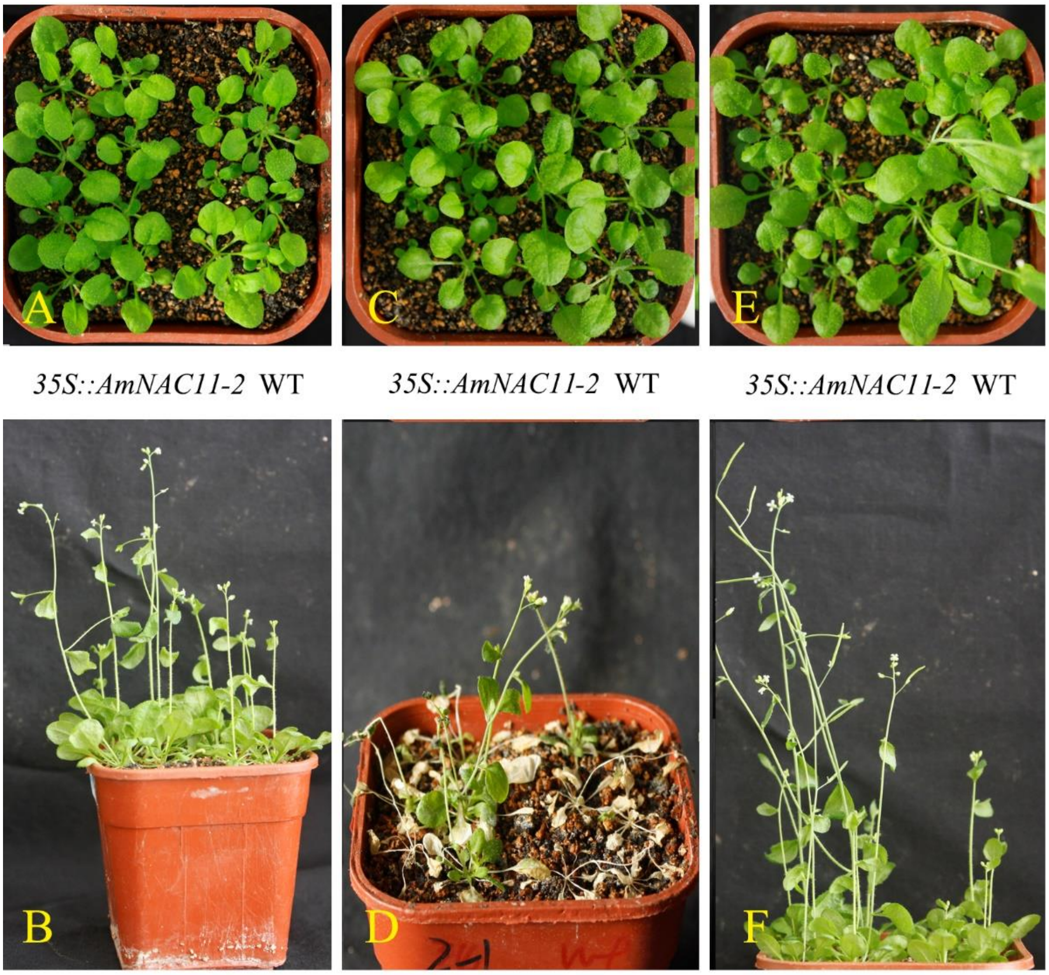

Figure S3 – Tolerance analyses of *AmNAC11* transgenic *Arabidopsis* plants ten days after cold stress.
