# Supplementary material for: Ammopiptanthus mongolicus stress-responsive NAC gene enhances the tolerance of transgenic Arabidopsis thaliana to drought and cold stresses
Source: Genet Mol Biol. 2019 Nov 14;42(3):624–34. doi: 10.1590/1678-4685-GMB-2018-0101 (PMC6905445; doi:10.1590/1678-4685-GMB-2018-0101)
Supplement: Supplementary file 4 [file 1415-4757-GMB-42-3-2018-0101-suppl4.pdf]

Supplementary Material to "*Ammopiptanthus mongolicus* stress-responsive *NAC* gene enhances the tolerance of transgenic *Arabidopsis thaliana* to drought and cold stresses"

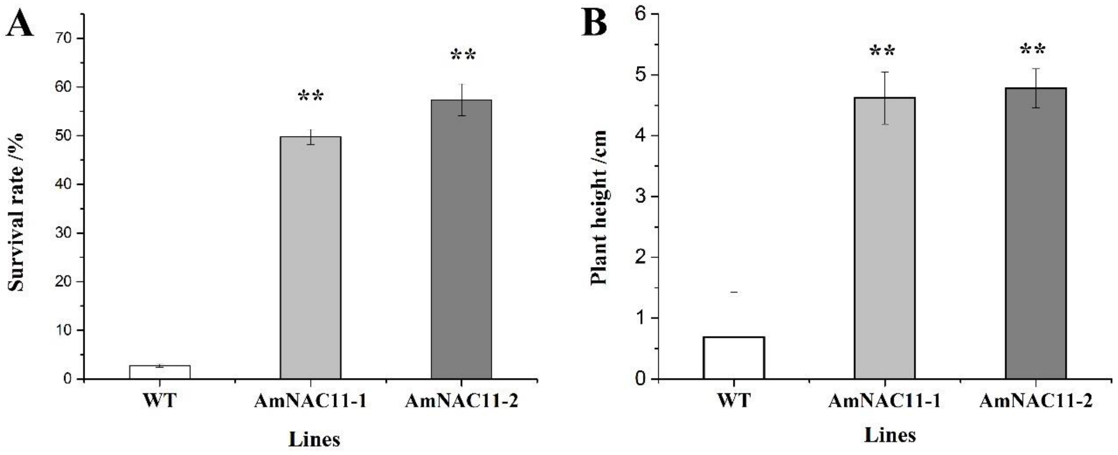

Figure S4 – Evaluation of cold resistance of *AmNAC11* transgenic *Arabidopsis* lines. (A) Survival rates of the wild type and transgenic lines on the 15th day after cold stress (** $p<0.05$); (B) Plant heights of the wild type and transgenic lines on the 15th day after cold stress (** $p<0.05$).
